# Draft genomes announcement of Vietnamese *Xanthomonas euvesicatoria* strains causing bacterial spot on pepper

**DOI:** 10.1099/acmi.0.000741.v3

**Published:** 2024-01-22

**Authors:** Aastha Subedi, Nguyen Thi Thu Nga, Doan Thi Kieu Tien, Gerald V. Minsavage, Pamela D. Roberts, Erica M. Goss, Jeffrey B. Jones

**Affiliations:** ^1^​ Department of Plant Pathology, University of Florida, Gainesville, Florida, USA; ^2^​ Department of Plant Protection, College of Agriculture, Can Tho University, Can Tho City, Vietnam; ^3^​ Southwest Florida Research & Education Center, University of Florida, Immokalee, Florida, USA; ^4^​ Emerging Pathogens Institute, University of Florida, Gainesville, Florida, USA

**Keywords:** bacterial-spot, pepper, Vietnam, whole-genome, *Xanthomonas*

## Abstract

*Xanthomonas euvesicatoria* the primary causal agent of bacterial spot of pepper (BSP), poses a significant global challenge, resulting in severe defoliation and yield losses for pepper growers. We present the whole genome sequences of eight *X. euvesicatoria* strains associated with BSP in Vietnam. These genomes contribute to representation of pepper production regions in the global sample of *X. euvesicatoria* genomes, enabling the development of precise global disease management strategies.

## Data Summary

Raw reads and the whole-genome assemblies have been deposited in GenBank (BioProject: PRJNA1035786). The accession numbers for type strains are *X*. *euvesicatoria* ATCC 11633 (JAJIUJ000000000) and *X*. *perforans* DSM-18975 (JABFFS000000000).

## Introduction

In the realm of agricultural sciences, the study of plant diseases and their causal agents holds paramount significance due to their far-reaching impacts on global food security. *Xanthomonas euvesicatoria*, a bacterium within the Xanthomonadaceae family, is one such pathogen that has necessitated considerable attention for a long time. This bacterium is well-known for causing bacterial spot disease in two economically important crops, tomatoes, and peppers [1–4]. The global significance of this pathogen emphasizes the need for in-depth genomic research to grasp its genetics and evolution, crucial for effective disease control strategies .

Vietnam, known for its diverse agricultural landscape, ranks as the seventh largest pepper producer in the Asia-Pacific region with production of 99 039 tons [5]. Approximately 75 % of Vietnam’s chillies pepper production is destined for international markets, securing its position as the second largest exporter of dry chillies, contributing over 18.6 % of the global exports in 2021 [6]. This production greatly contributes to the country’s agricultural exports and strengthens the economic prosperity of Vietnam. Thus, there is a need to address economically damaging diseases such as BSP, particularly when considering the lack of genomic data available for *Xanthomonas* strains affecting pepper in Vietnam.

In this study, we present a draft genome announcement of *X. euvesicatoria* isolated from Vietnam. This draft genome announcement serves as a preliminary report on the genomic information for *X. euvesicatoria* from Vietnam, facilitating in-depth studies on its biology, epidemiology, and regional diversity. Further research using these genomes can provide useful information when developing disease management strategies in Vietnam and beyond.

## Methods

In 2013, symptomatic pepper leaves from eight different locations including five provinces/cities of Vietnam were collected. Sections of individual lesions from a single leaf were macerated in 25 µl of sterile tap water, streaked on Nutrient agar (NA) in a quadrant pattern, and then incubated at 28 °C for 3–5 days to isolate single colonies. Pathogenicity and pepper race determinations in pepper differentials were determined as explained in Subedi *et al*. [4]. Briefly, the pepper cultivar 'Early California Wonder' (ECW) and its near-isogenic lines carrying resistance genes (*Bs1*, *Bs2*, *Bs3*) were infiltrated with bacterial suspensions of 10^8^ c.f.u. ml^−1^. The infiltrated leaves were observed for hypersensitive or susceptible responses at five time points (12, 24, 36, 48, and 72 hpi). Strains were categorized into races based on responses in the differential lines, with susceptibility confirmed in ECW [7]. Strains were also assessed for amylolytic activity by streaking bacterial cells from 24 h cultures grown on NA onto 1.5 % soluble starch-amended NA plates and incubating at 28 °C for 48 h. Amylase activity was determined by observing the presence of a turbid halo around each colony.

Pure cultures of isolated strains preserved at −80 °C were streaked on NA and incubated at 28 °C. Fresh bacterial cultures after 24 h growth were transferred into test tubes containing nutrient broth and incubated overnight at 28 °C on a shaker set at 250 r.p.m. Genomic DNA was extracted from overnight nutrient broth cultures using the Wizard Genomic DNA Purification Kit (Promega, Madison, WI) for Gram-negative bacteria. The extracted DNA was sent to Seq Centre (Pittsburgh, PA) and sequenced on the Illumina Next-Seq2000 platform, yielding 151 bp paired-end reads. Illumina DNA Prep kit and IDT 10 bp UDI indices were used to prepare sample libraries. The bcl-convert (v3.9.3), a proprietary Illumina software, was employed for demultiplexing, quality control, and adapter trimming. Raw reads were assembled following the procedures described in Timilsina *et al*. [8]. Unless otherwise noted, default parameters were used for all software. Initially, TrimGalore (v.0.6.10) was employed to remove adapters [9]. Subsequently, SPAdes (v.3.10.1) with ‘careful’ switch was used for assembly [10], discarding <500 bp contigs and a K-mer coverage of <2.0. Bowtie 2 (v. 2.3.3) was used to align the validated reads (reads obtained after removing adpators using TrimGalore) with filtered contigs [11]. SAMtools (v.1.18) was used to convert SAM files to BAM files [12]. Pilon (v.1.24) with ‘frags’ setting was applied to refine the draft genome assemblies [13] and polishing was applied only one round. Genome statistics were computed through a python script (https://github.com/sujan8765/nepgorkhey_python/blob/master/genome_stats.py). GC content of assembled whole genome was obtained using perl script (https://github.com/valdeanda/Useful_scripts/blob/master/get_gc_content.pl). Pyani (v.0.2.10) with ‘ANIb’ was employed to determine the average nucleotide identity (ANI) among assemblies and compare them to type strains of *X. euvesicatoria* ATCC 11633 and *X. perforans* DSM-18975 [14]. Prokaryotic Genome Annotation Pipeline v.6.4 provided from the National Centre for Biotechnology Information was used to annotate the genomes [15].

## Results and discussion

All the strains were pathogenic on pepper cultivar ECW and were identified as pepper race P1. The prevalence of race P1 restricts the utilization of *Bs1* resistance gene, but there remains a promising opportunity to harness the effectiveness of other commercially available dominant resistant genes, *Bs2* and *Bs3* for the management of BSP in Vietnam. Prior to the availability of sequencing technology, one of the techniques for differentiation of *Xanthomonas* species causing bacterial spot in tomato and pepper involved assessing their amylolytic activity. In previous studies *X. euvesicatoria* strains were either negative or weakly amyloytic [1, 7]; however, in another study a few *X. eucvesicatoria* strains from Mexico and from Ohio in the USA had amylolytic activity [1, 16]. In this study, all strains from Vietnam were non-amylolytic, which is typical of *X. euvesicatoria* strains. However, there is an increasing number of reports indicating the presence of amylolytic *X. euvesicatoria* strains [4, 17–19]. This finding highlights the need for a more extensive and comprehensive study, incorporating a larger and more varied sample sizes, to better understand the pathogen dynamics.

**Fig. 1.**
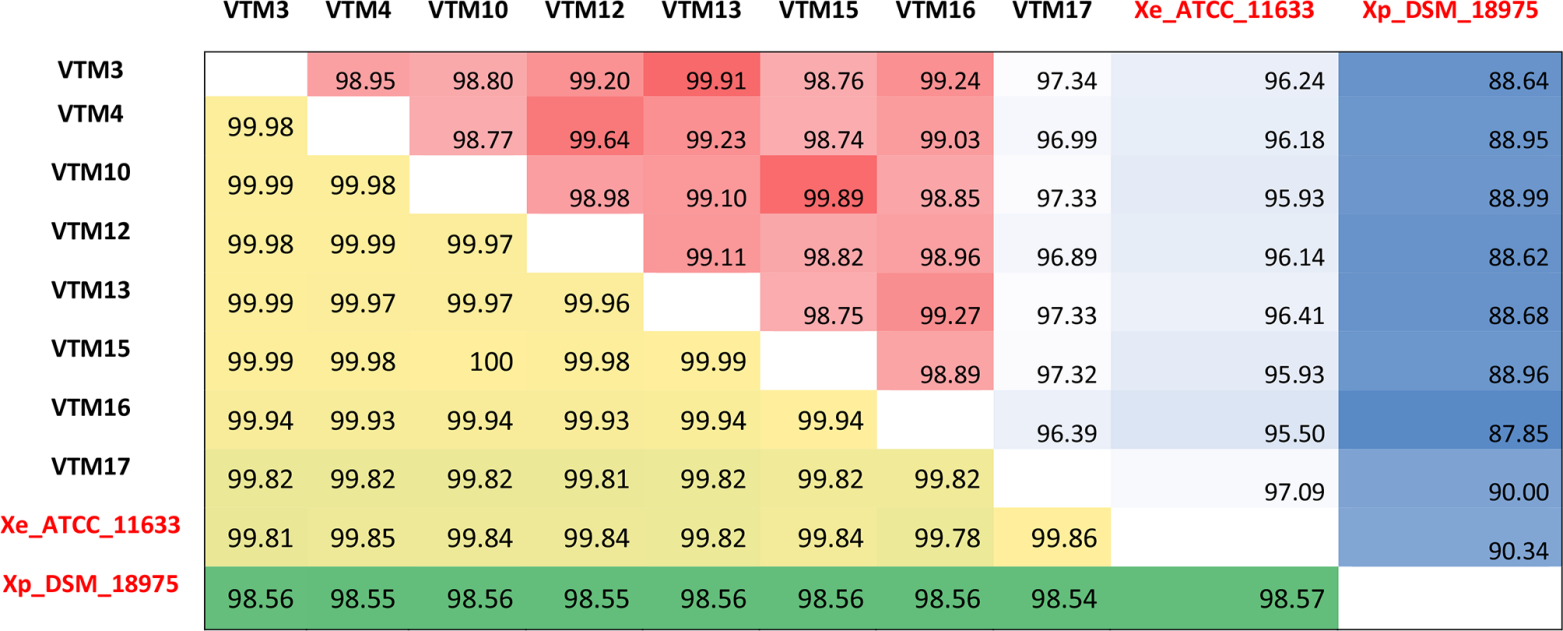
Pairwise average nucleotide identity and alignment coverage shown in percentage between the sequenced *Xanthomonas euvesicatoria* strains and with type strains – *Xanthomonas euvesicatoria* ATCC 11633 and *Xanthomonas perforans* DSM-18975. The type strains are shown in red.

Genome analysis of eight Vietnamese strains revealed average genome lengths of 5.18 Mbp ranging from 5.12 to 5.24 Mbp, with an average contig count of 88 (range=65 to 95) and an average sequencing coverage of 62 (range=56 to 70). The average N50 of all genomes was 193 424 bp (Table 1) . Whole-genome average nucleotide identity (ANI) analysis confirmed that all sequenced Vietnam strains belonged to *X. euvesicatoria*, with >99.78 % ANI compared to the *X. euvesicatoria* type strain ATCC 11633 and <98.54 % ANI compared to the *X. perforans* type strain DSM_18975 (Fig. 1). The genomic similarity among the sequenced strains varied from 99.81–100 %. Annotation by NCBI predicted 95 RNA genes and 52 tRNA genes in all sequenced strains except VTM17 (RNA genes=94; tRNA genes=51). The number of predicted protein-coding genes ranged from 4189 to 4324 (average=4265).

**Table 1.** Genome statistics of Vietnamese *Xanthomonas euvesicatoria* strains isolated from pepper along with type strains of *Xanthomonas euvesicatoria* ATCC 11633 and *Xanthomonas perforans* DSM-18975

Strain Designation	Isolation location	Species	Number of Contigs	Genome Length (bp)	Genome Coverage(X)	N50(bp)	RNA genes	tRNAs	CDSs	Total Raw reads Pairs	GC content (%)	Accession Number	SRA
VTM3	Thanh Binh district, Dong Thap Province	*X. euvesicatoria*	94	5 198 942	65	222 496	95	52	4278	1138617	64.63	JAWUDP000000000	SRR26670405
VTM4	Binh Tan didtrict, Vinh Long Province	*X. euvesicatoria*	89	5 176 495	63	167 086	95	52	4250	1095487	64.65	JAWUDQ000000000	SRR26670404
VTM10	My An, Long Xuyen city, An Giang province	*X. euvesicatoria*	90	5 172 799	56	193 817	95	52	4253	978 900	64.63	JAWUDR000000000	SRR26670403
VTM12	My Thuan, Long Xuyen city, An Giang province	*X. euvesicatoria*	95	5 193 250	62	167 064	95	52	4290	1087075	64.63	JAWUDS000000000	SRR26670402
VTM13	Thanh Hoa, Chau Thanh district, An Giang province	*X. euvesicatoria*	88	5 197 259	62	200 665	95	52	4282	1087855	64.63	JAWUDT000000000	SRR26670401
VTM15	Co Do, O Mon district, Can Tho city	*X. euvesicatoria*	94	5 172 604	57	163 170	95	52	4261	996 669	64.63	JAWUDU000000000	SRR26670400
VTM16	Long Hung, O Mon district, Can Tho city	*X. euvesicatoria*	93	5 244 360	70	210 776	95	52	4324	1250327	64.66	JAWUDV000000000	SRR26670399
VTM17	Chau Thanh, Hau Giang province	*X. euvesicatoria*	65	5 122 930	63	222 315	94	51	4189	1085621	64.67	JAWUDW000000000	SRR26670398
Type Strain ATCC 11633	USA: New Jersey	*X. euvesicatoria*	121	5 314 92 2	82	101 995	-	52	4320	1491054	64.5	JAJIUJ000000000	SRR16936533
Type Strain DSM-18975	USA	*X. perforans*	3	5 034 53 2	435	4914073	94	53	4138	549 782	65	JABFFS000000000	SRR11788032

Despite the relatively limited number of samples and the absence of apparent phenotypic diversity, ANI revealed a degree of genetic variation among *X. euvesicatoria* strains in Vietnam. For instance, VTM17 stands out from other strains with its lower ANI of 99.8 % setting it apart from the rest, which have ANIs of over 99.9 %. This genetic diversity might be linked to other factors, including the presence of specific pathogenic or virulence-related genes in the strains and the potential antibiotic or bactericide resistance genes. This emphasizes how genetic analysis can reveal subtle differences within the populations, even when visible traits don't provide clear distinctions. Deeper investigations, incorporating genomes from around the world, will provide insights into their genetic relationships with other strains and populations, including variation in effectors. This knowledge can be harnessed for the development of targeted strategies for global disease management.

## References

[1] Jones JB, Lacy GH, Bouzar H, Stall RE, Schaad NW (2004). Reclassification of the xanthomonads associated with bacterial spot disease of tomato and pepper. Syst Appl Microbiol.

[2] Klein-Gordon JM, Xing Y, Garrett KA, Abrahamian P, Paret ML (2021). Assessing changes and associations in the *Xanthomonas* perforans population across Florida commercial tomato fields via a statewide survey. Phytopathology.

[3] Potnis N, Timilsina S, Strayer A, Shantharaj D, Barak JD (2015). Bacterial spot of tomato and pepper: diverse *Xanthomonas* species with a wide variety of virulence factors posing a worldwide challenge. Mol Plant Pathol.

[4] Subedi A, Minsavage GV, Jones JB, Goss EM, Roberts PD (2023). Exploring diversity of bacterial spot associated *Xanthomonas* population of pepper in Southwest Florida. Plant Dis.

[5] Food and Agriculture Organization of the United Nations (FAO) (2021). FAOSTAT statistics database.

[6] (2021). ITC Trade Map. https://www.trademap.org.

[7] Bouzar H, Jones JB, Stall RE, Hodge NC, Minsavage GV (1994). Physiological, chemical, serological, and pathogenic analyses of a worldwide collection of *Xanthomonas campestris* pv. *vesicatoria* strains. Phytopathology.

[8] Timilsina S, Pereira-Martin JA, Minsavage GV, Iruegas-Bocardo F, Abrahamian P (2019). Multiple recombination events drive the current genetic structure of *Xanthomonas perforans* in Florida. Front Microbiol.

[9] Martin M (2011). Cutadapt removes adapter sequences from high-throughput sequencing reads. EMBnet j.

[10] Prjibelski A, Antipov D, Meleshko D, Lapidus A, Korobeynikov A (2020). Using SPAdes De Novo assembler. Curr Protoc Bioinformatics.

[11] Langmead B, Salzberg SL (2012). Fast gapped-read alignment with Bowtie 2. Nat Methods.

[12] Li H, Handsaker B, Wysoker A, Fennell T, Ruan J (2009). The sequence alignment/map format and SAMtools. Bioinformatics.

[13] Walker BJ, Abeel T, Shea T, Priest M, Abouelliel A (2014). Pilon: an integrated tool for comprehensive microbial variant detection and genome assembly improvement. PLoS One.

[14] Pritchard L, Cock P, Esen Ö (2019). pyani v0. 2.8: average nucleotide identity (ANI) and related measures for whole genome comparisons.

[15] Tatusova T, DiCuccio M, Badretdin A, Chetvernin V, Nawrocki EP (2016). NCBI prokaryotic genome annotation pipeline. Nucleic Acids Res.

[16] Bouzar H, Jones JB, Somodi GC, Stall RE, Daouzli N (1996). Diversity of *Xanthomonas campestris* pv. vesicatoria in tomato and pepper fields of Mexico. Can J Plant Pathol.

[17] Hamza AA, Robène-Soustrade I, Boyer C, Laurent A, Jouen E (2010). A new type of strain of *Xanthomonas euvesicatoria* causing bacterial spot of tomato and pepper in Grenada. Plant Dis.

[18] Osdaghi E, Taghavi SM, Hamzehzarghani H, Lamichhane JR (2016). Occurrence and characterization of the bacterial spot pathogen *Xanthomonas euvesicatoria* on pepper in Iran. J Phytopathol.

[19] Stoyanova M, Vancheva T, Moncheva P, Bogatzevska N (2014). Differentiation of *Xanthomonas* spp. Causing bacterial spot in Bulgaria based on biolog system. Int J Microbiol.

